# Israeli nurse practice environment characteristics, retention, and job satisfaction

**DOI:** 10.1186/2045-4015-3-7

**Published:** 2014-02-24

**Authors:** Freda DeKeyser Ganz, Orly Toren

**Affiliations:** 1 Henrietta Szold Hadassah Hebrew University, School of Nursing at the Faculty of Medicine, P.O. Box 12000, Jerusalem, Israel; 2 Safety and Risk Management, Hadassah Medical Organization, Ein Kerem, Jerusalem, Israel

**Keywords:** Practice environment, Job satisfaction, Nurse retention, Israel

## Abstract

**Background:**

There is an international nursing shortage. Improving the practice environment has been shown to be a successful strategy against this phenomenon, as the practice environment is associated with retention and job satisfaction. The Israeli nurse practice environment has not been measured. The purpose of this study was to measure practice environment characteristics, retention and job satisfaction and to evaluate the association between these variables.

**Methods:**

A demographic questionnaire, the Practice Environment Scale, and a Job Satisfaction Questionnaire were administered to Israeli acute and intensive care nurses working in 7 hospitals across the country. Retention was measured by intent to leave the organization and work experience. A convenience sample of registered nurses was obtained using a bi-phasic, stratified, cluster design. Data were collected based on the preferences of each unit, either distribution during various shifts or at staff meetings; or via staff mailboxes. Descriptive statistics were used to describe the sample and results of the questionnaires. Pearson Product Moment Correlations were used to determine significant associations among the variables. A multiple regression model was designed where the criterion variable was the practice environment. Analyses of variance determined differences between groups on nurse practice environment characteristics.

**Results:**

610 nurses reported moderate levels of practice environment characteristics, where the lowest scoring characteristic was ‘appropriate staffing and resources’. Approximately 9% of the sample reported their intention to leave and the level of job satisfaction was high. A statistically significant, negative, weak correlation was found between intention to leave and practice environment characteristics, with a moderate correlation between job satisfaction and practice environment characteristics. ‘Appropriate staffing and resources’ was the only characteristic found to be statistically different based on hospital size and geographic region.

**Conclusions:**

This study supports the international nature of the vicious cycle that includes a poor quality practice environment, decreased job satisfaction and low nurse retention. Despite the extreme nursing shortage in Israel, perceptions of the practice environment were similar to other countries. Policy makers and hospital managers should address the practice environment, in order to improve job satisfaction and increase retention.

## Introduction

The nursing shortage is an international problem and has been shown to negatively affect healthcare systems and patient outcomes worldwide [1]. It has been associated with many different causes, including an inability of the healthcare system to retain nurses [2], due in part to a lack of nurses willing to work under the current working conditions [1]. The nurse practice environment has been found in other countries to be an important factor associated with recruitment, retention and job satisfaction [3-5]. This issue takes on greater importance in an environment of workforce shortages where an attractive work environment is thought to act like a magnet for nurse recruitment and retention [6,7], attracting workers, while a poorer environment many be associated with a lack of job satisfaction and increased intent to leave the organization. This topic is therefore of significance in that it will help provide information that might ensure care delivery in hospitals with workforce shortages. The current study describes practice environment characteristics and their association with nurse retention and job satisfaction in acute care hospitals in Israel.

## Background

### Nursing and the Israeli healthcare system

Nursing in Israel is regulated by the Nursing Division of the Ministry of Health, whose role is to initiate and supervise national nursing policy, including forecasting and planning nursing manpower needs. Almost all of the nurses are unionized under one national nurses’ union. Salaries and other benefits do not differ between organizations because they are based on national agreements signed by the nurses’ union. Therefore there is no direct incentive for a nurse to move from one healthcare institution to another based on salary or benefits. This situation might positively affect the level of nurse retention in Israel. However, the number of Israeli nurses has plummeted each year to a low of 4.97 nurses per 1000 population, despite efforts by the Ministry of Health to increase the number of nursing students [8], and the fact that most registered nurses are working as nurses (89%) in full time positions (67%) [9]. These system characteristics can affect the nurse practice environment, job satisfaction and retention.

### The nurse practice environment

The practice environment is defined as the organizational characteristics of the work environment that help or hinder professional nursing practice [10,11]. It is divided into five characteristics: 1. participation of nurses in the management of the hospital, 2. the nursing foundation for quality care, 3. nursing administration capability, leadership and support of nurses, 4. staffing and appropriate use of resources, 5. and relationships between physicians and nurses. The nurse practice environment has been studied in many different nurse populations including nurses in out-patient, acute and intensive care and in several countries including the United States, Australia, Canada, Iceland and Taiwan [12], Estonia [13], Finland and Holland [14], and Ireland [15]. Consistent findings between countries were found in a review of the use of the Practice Environment Scale (PES), a popular measure of the nurse practice environment, while differences existed on what subscale was rated highest [12].

### Nurse retention/turnover

Many countries are troubled over the problem of nurse retention and turnover and how to solve it [16]. For example, in the US, it has been reported that on average 8.5-14% of nurse positions are vacant, and that these rates were higher in internal medicine and critical care units [17]. Nirel and colleagues [9] found that Israeli medicine units had the highest turnover levels and that 48% of intensive care nurses transferred from medicine units. An increase in the number of nurses interested in working outside of the country in the last 10 years was also reported [18,19], including 286 other nurses that requested the paperwork to work outside of Israel. According to an article in the local press at that time [20], the reasons behind the intent to leave were lack of a sense of autonomy; lack of staffing that leads to overwork; poor quality of care; and the performance of non- professional tasks, characteristics similar to the practice environment.

In a review of the nurse practice environment, it was reported that several studies found significant negative correlations between the practice environment and intent to leave [12]. A more recent systematic review of 39 studies found that improving the nurse practice environment was an important strategy to improve nurse retention [16].

Taiwanese nurses who had better perceptions of their practice environment were less likely to report their intentions to leave their place of employment [21]. However, a study of East Caribbean nurses working in in-hospital units [22] found that practice environment scores were not correlated with reported intent to leave in 2 or 5 years.

Intent to stay at the current place of employment was significantly correlated with the practice environment for Jordanian ICU nurses but not for acute care nurses [23].

### Job satisfaction

Working conditions and the organizational environment were found to be significantly associated with nurse job satisfaction as found in a systematic review of 100 papers related to nurse job satisfaction [24]. Manojlovich and Laschinger [25] conclude that there is considerable evidence that job satisfaction is linked to nursing practice environments. Job satisfaction was found to be a consistent underlying component associated with workplace factors that was associated with nursing job turnover [26]. Van Bogaert and colleagues [27] found that physician-nurse relationships and unit management were significantly associated with job satisfaction among a sample of acute and critical care nurses in Belgium. This finding was also summarized by Warshawsky and & Havens [12] in their review of the practice environment literature. Nirel and colleagues [9] found that 69% of Israeli hospital nurses were satisfied with their job as opposed to 79% of nurses who work in the community. It was also found that nurses who reported having higher workload levels were less satisfied with their job.

The American Association of Critical Care Nurses [28] has promoted the concept of "healthy work environments", defined as work places with organizational structures that are dedicated to achieving the goals of the organization and nurse job satisfaction. Six standards were defined that could be used to develop a healthy work environment that include skilled communication, trustworthy leadership, significant recognition, appropriate staffing, effective decision making and collegial collaboration between workers [29].

In summary, the international literature has shown that there is an association between characteristics of the practice environment, nurse retention and job satisfaction. This subject has yet to be investigated in Israel. Therefore the objectives of this study were to describe the practice environment, nurse retention and job satisfaction among Israeli registered nurses working in acute and intensive care units; and to determine whether there is a relationship among these factors. In this way, areas that are in need of improvement could be identified and measures can be taken to improve these areas.

## Method

### Design

This was a cross-sectional, descriptive, correlational study.

### Sample

A convenience sample of registered nurses was obtained using a bi-phasic, stratified, cluster design. In the first phase, hospitals were divided into one of eight categories based on geographic region [north, central, south and Jerusalem] and size [medium: 300-600 beds or large: over 601 beds]. There are no small hospitals in Israel. An attempt was made to choose at least one hospital from each of the eight categories. In the second phase, hospital units (or clusters) were chosen. All general intensive care units from the designated hospitals were included while medicine units (thought to be representative of acute care) were randomly chosen. All of the registered nurses who worked on the chosen units were asked to participate.

### Data collection tools

Three tools were used: the Practice Environment Scale [10]; a demographic and professional characteristics questionnaire and the Hadassah Medical Organization Nurse Job Satisfaction Questionnaire.

#### Practice Environment Scale (PES)

The Practice Environment Scale (PES) was developed to measure the nurse practice environment [10]. It contains 31questions in a Likert format. Responses were in the range from 1 (strongly disagree) to 4 (strongly agree). The questionnaire is based on the Nursing Work Index (NWI) and the Nursing Work Index- Revised that measured magnet hospital characteristics [30,31]. After completing a factor analysis of the NWI data, the PES was developed and is comprised of 5 characteristics: nurse participation in hospital affairs; nursing foundations for quality of care; nurse manager ability, leadership, and support of nurses; staffing and resource adequacy; and collegial nurse-physician relations. Subscale scores were determined as the mean of all items in that subscale while the total PES score was the total item mean. Developers of the PES report Cronbach alpha levels of .71-.84 for the entire scale and its components in large samples of American and Canadian nurses [10,11]. Evidence of the tool’s validity was also demonstrated in several studies [10,11,31].

#### Demographic and professional characteristics questionnaire

The following characteristics were measured: age, sex, family status, religion (measure of ethnicity in Israel), type of hospital unit (internal medicine or intensive care), level of nursing education (registered nurse, registered nurse with BA, registered nurse with masters’ degree or higher), post basic certification, current nursing role and years of experience as a nurse, in the current institution and in the current unit.

#### Nurse retention

Nurse retention was measured in two ways: a. nursing employment experience (in the current institution and the current unit) and by the following question, "I intend to leave my workplace in the next 12 months", measured on a scale from 1 (do not intend at all) to 5 (definitely intend). This single item has been used in several previous studies and has been found to adequately measure retention [32-34].

#### Job satisfaction

Job satisfaction was measured in two ways. The first was using the Nurse Job Satisfaction Questionnaire of the Hadassah Medical Organization. This unpublished questionnaire was designed by a panel of nurse experts at the Hadassah Medical Organization who based their choice of items from the literature. The questionnaire underwent several revisions and two rounds of factor analysis. The final version used in this study is composed of 25 questions on a Likert scale ranging from 1 (strongly agree) to 5 (strongly disagree). The questionnaire is divided into seven sections: relationships with superiors, characteristics of the role, interpersonal relationships, autonomy, stress, feelings towards the organization and work challenges. The tool has demonstrated internal reliability (Cronbach alpha = .93 for the entire questionnaire and .71-.86 for each section). Job satisfaction was also measured using a single question that measures overall job satisfaction, "To what extent are you happy with your place of employment?" measured on a range from 0 to 5.

### Data collection

After receiving administration and ethical approval from each institution, a pilot test was conducted and determined that the method of data collection was sound.

Data were collected based on the preferences of each unit. In some units, questionnaires were distributed during various shifts while the research assistant was present while others filled out their questionnaires during staff meetings. In other units, questionnaires were left in staff mailboxes and reminders were placed to improve the level of questionnaire return. In this case, questionnaires were returned in sealed envelopes to a central location on the unit. Data were anonymous.

### Statistical analysis

Descriptive statistics (means, standard deviations and frequencies) were used to describe the sample and results of the questionnaires. Pearson Product Moment Correlations were used to determine significant associations among the variables.

When bivariate relationships between the variables were found to be significant, a multiple regression model was designed where the criterion variable was the nurse practice environment. Analyses of variance were also conducted to determine differences between groups on nurse practice environment characteristics. Internal consistency reliability was measured using Cronbach’s alpha. Data were analyzed using SPSS version 17.

## Results

The sample consisted of 610 nurses from 7 hospitals across the country. The response rate ranged from 63-73%, depending on hospital and unit. Most of the respondents were Jewish (n = 452, 77.8%), married (n = 423, 71.7%), women (n = 468, 78.5%) who were born in Israel (n = 285, 51.4%). Mean age was 38.3 (*SD =* 8.9) (Table 1). The mean level of practice environment characteristics was moderate (2.73 + 0.43, out of 4). Mean scores for the components of the practice environment ranged from 2.47 (nurse participation in hospital affairs) to 3.00 (collaboration between physicians and nurses) (Table 2). Mean nursing experience was 13.1 + 8.8 years, 11.3 + 8.3 at the current institution and 9.2 + 9.2 on the current unit. Mean intent to leave was 1.79 + 1.11, out of 5. The level of job satisfaction as measured by the questionnaire was moderate/high (3.87 + 0.59, out of 5). Responses to the single question about overall job satisfaction were 3.81 + 0.84 (out of 5) (Table 2).

**Table 1:** Demographic and professional characteristics (N = 610)

**Personal characteristics**		**n**	**%**	**Professional characteristics**		**n**	**%**
**Gender:**	Female	468	78.5	**Educational level:**	RN	127	21.6
	Male	128	21.5		BA to RN	41	7.0
	Missing	14			Generic RN	324	55.0
					RN to BSN	84	14.3
**Age (years):**	19-29	102	18.4		Other	13	2.2
	30-39	227	40.9		Missing	21	
	40-49	162	29.2				
	50-59	55	9.9	**Post basic certification**:	Yes	296	51.7
	60+	9	1.6		No	277	48.3
	Missing	56			Missing		37
**Marital status:**	Single	109	18.5	**Nursing role**:	Staff nurse	509	86.0
	Married	423	71.7		Asst head/Head nurse	50	8.4
	Divorced	54	9.2		Instructor	24	4.1
	Widowed	4	0.7		Manager/Supervisor	1	0.2
	Missing	20			Other	8	1.4
					Missing	18	
**Religion:**	Jewish	452	77.8				
	Moslem	81	13.9	**Employment status**:	Full time	486	83.4
	Christian	32	5.5		Part time	104	7.6
	Other	16	2.8		Missing	20	
	Missing	29					
**Place of birth:**	Israel	255	51.4				
	Former USSR	243	43.8				
	W. Europe/ N. America	17	3.1				
	Africa	10	1.8				
	Missing	85					

**Table 2:** PES characteristics, level of nurse retention and job satisfaction

**Variable**	**n**	**Mean**	**Standard deviation**
**PES characteristics**			
Nurse participation in hospital affairs	583	2.61	0.57
Staffing and resource adequacy	583	2.85	0.46
Nursing foundations for quality of care	583	2.91	0.52
Nurse manager ability, leadership, and support of nurses	583	3.00	0.58
Collegial nurse-physician relations	583	2.73	0.43
Total	583	2.47	0.57
**Retention**			
Tenure in the current unit (years)	587	9.20	7.6
Intention to leave (scale from 1-5)	590	1.79	1.11
**Job satisfaction**			
Job satisfaction questionnaire (scale from 1-5)	599	3.87	0.59
General job satisfaction (1 question, scale from 1-5)	610	3.81	0.84

A statistically significant negative weak correlation was found between all of the PES characteristics and intent to leave while a moderate positive correlation was found between all of the characteristics and job satisfaction (Table 3).

**Table 3:** Pearson correlations between PES characteristics, nurse retention and job satisfaction (n = 577)

**PES characteristic**	**Years in organization**	**Years on unit**	**Intention to leave**	**General job satisfaction**
Nurse participation in hospital affairs	-.008	.016	-.277**	.523**
Staffing and resource adequacy	.119**	.125**	-.285**	.451**
Nursing foundations for quality of care	.119**	.125**	-.290**	.563**
Nurse manager ability, leadership, and support of nurses	-.009	-.008	-.269**	.528**
Collegial nurse-physician relations	-.055	.034	-.268**	.464**
Total	.059	.081	-.347**	.644**

**p < .01.

Four variables were placed into a regression model where the mean PES score was used as the criterion variable and place of employment (acute or intensive care); intent to leave, mean satisfaction score and total years as a nurse were the predictor variables. While the overall model was significant (R = .72, p < .001), only job satisfaction was found to significantly contribute to the model (t = 17.0, p < .001). ‘Appropriate staffing and resources’ was the only practice environment characteristic found to be statistically different based on geographic region (F(3,558) = 4.49, p = .004) (Table 4) and hospital size (t(594) = 3.76, p < .001) (Table 5). Based on a Bonferroni post hoc analysis, it was found that hospitals in the north were significantly different from the central region. Medium sized hospitals scored higher than large hospitals for this characteristic. All other comparisons were not found to be significant.

**Table 4:** PES characteristics by geographic region (N = 583)

**PES characteristic**	**Northern region**	**Central region**	**Jerusalem**	**Southern region**
	***M***	***M***	***M***	***M***
	***(SD)***	***(SD)***	***(SD)***	***(SD)***
Nurse participation in hospital affairs	2.46	2.46	2.51	2.47
	*(0.62)*	(0.57)	(0.53)	(0.57)
Staffing and resource adequacy*	2.74	2.54	2.62	2.67
	(0.54)	(0.57)	(0.59)	(0.48)
Nursing foundations for quality of care	2.84	2.85	2.84	2.86
	(0.51)	(0.44)	(0.47)	(0.65)
Nurse manager ability, leadership, and support of nurses	2.90	2.89	2.92	3.06
	(0.58)	(0.52)	(0.53)	(0.51)
Collegial nurse-physician relations	3.10	2.98	2.97	2.97
	(0.60)	(0.55)	(0.59)	(0.66)
Total	2.46	2.72	2.74	2.77
	(0.62)	(0.42)	(0.44)	(0.43)

*p < .01.

**Table 5:** PES characteristics by hospital size (N = 583)

**PES characteristic**	**Medium**	**Large**
	***M***	***M***
	***(SD)***	***(SD)***
Nurse participation in hospital affairs	2.49	2.46
	(0.60)	(0.55)
Staffing and resource adequacy*	2.74	2.55
	(0.53)	(0.57)
Nursing foundations for quality of care	2.90	2.83
	(0.48)	(0.45)
Nurse manager ability, leadership, and support of nurses	2.94	2.89
	(0.54)	(0.51)
Collegial nurse-physician relations	3.07	2.97
	(0.63)	(0.55)
Total	2.79	2.72
	(0.46)	(0.43)

*p < .01.

## Discussion

The results of this study lend further evidence to the relationship between the practice environment, job satisfaction and retention. The best predictor for perceived practice environment was job satisfaction while ‘adequate resources and staffing’ was the only characteristic found to significantly differ between hospital size and geographic region.

The most significant and lowest rated characteristic of the nursing practice environment was ‘appropriate staffing and resources’. Warshawsky and Havens [12] found the same result in their review of several studies investigating the nurse practice environment. These results are not surprising given the fact that current occupancy rates of general acute care, especially internal medicine units, in Israel are very high.

Nirel and colleagues [9] found the highest level of nursing staff turnover in Israel was from general internal medicine units to specialty units. These conditions are exacerbated by a lack of increase in the number of nursing staffing positions since 1996 that are not appropriate for the current conditions [35]. These findings demonstrate that this aspect of the work environment necessitates change. In a review of the nursing practice environment of acute care nurses, Garon and Ringle [36] concluded that the majority of studies examined found a significant relationship between working conditions, workload, staffing, and job satisfaction. These findings are echoed in the current study.

Retention was measured using two different methods–intent to leave and nurse work experience. The level of intent to leave was found to be relatively low (8.8%). Internationally, there is a rather large range of reported intent to leave (13.5-67.5%) [37,38]. In the only other Israeli study that investigated intent to leave, only 5% reported intent to leave within the next year with another 6% reporting such intentions in the long term [9].

Intent to leave was found to be significantly associated with the practice environment. Similar results were found in western [38,39] and Eastern Europe [40], the US [41], Lebanon [37,42], and Taiwan [43]. On the other hand, no significant relationships were found with any of the practice environment characteristics and any of the measures of nurse work experience. It seems possible that other intervening variables may be responsible for this negative finding. One possible factor is that nursing working conditions in Israel are determined on a national level via direct negotiations between the Ministry of Health and the Nurses’ Union. This includes the nurse patient ratio as well as salary and other work benefits. There is no variability in salary conditions between hospitals. Therefore, individual nurses are less likely to change their place of employment for improved working conditions. This aspect of the Israeli health care system might also explain the lack of differences between acute and critical care units with respect to intent to leave and tenure in the current place of employment. Another possible intervening variable is the economic situation. Data were collected for this study during the international economic downturn. During this time period some hospitals were cutting back on their budgets and were not hiring new nurses.

Job satisfaction was found to be moderately high with moderate correlations between job satisfaction measures and the nurse practice environment. Similar results were shown among nurses in Taiwan [43] and Europe [39].

### Study limitations

The study involved only seven hospitals and two types of units. These units were chosen because it was assumed that they represented the extremes of the practice environment. This did not turn out to be the case and perhaps other units should have been chosen instead. Another limitation is the moderate response rate (63-73%). Despite the fact that many of the analyses were found to be statistically significant, the actual numerical difference was small between groups and might not be of practical or clinical significance. This statistical result is possible due to the large sample size.

### Recommendations

Given the fact that the nursing shortage is expected to worsen, policy makers and nursing administrators should acknowledge and try to improve those practice environment characteristics that were shown to be related to nurse job satisfaction and intent to leave. Special attention should be paid to staffing and resource allocation in larger hospitals and in the central region of the country. Other variables impacting on the environment should also be investigated, such as secondary post-traumatic stress disorder, burnout, and nurse empowerment. The development of programs to improve the nurse practice environment, especially staffing and resources, could improve nurse retention and thereby slow down the nursing shortage.

## Conclusions

Nurses in Israel rate their practice environment as moderate. The most significant and lowest rated characteristic was ‘appropriate staffing and resources’. This characteristic consistently differed between types of units, geographic region and hospital size. Results showed that while job satisfaction and intent to leave were associated with the practice environment, nursing experience was not. It would seem that other factors impact on nurses’ intentions to leave their work place.

Given the fact that the nursing shortage is expected to worsen, policy makers and nursing administrators should acknowledge and try to improve those practice environment characteristics shown to be related to nurse job satisfaction and intent to leave. Special attention should be paid to staffing and resource allocation. Other variables impacting on the environment should also be investigated. The development of programs to improve the nurse practice environment, especially staffing and resources, could slow down the nursing shortage. These programs should be especially directed towards specific types of hospitals, units, and geographic regions.

## Competing interests

The authors declare that they have no competing interests.

## Authors’ contributions

FDG and OT initiated, planned, and implemented this study. FDG wrote the manuscript and OT reviewed it and gave her final approval. Both authors read and approved the final manuscript.

## Authors’ information

FDG is an Associate Professor of Nursing at the Henrietta-Szold Hadassah Hebrew University School of Nursing, Faculty of Medicine, Jerusalem, and is currently serving as Coordinator of Research and Development.

OT lecturers at the Henrietta-Szold Hadassah School of Nursing and at the School of Public Health, Hebrew University, Jerusalem, and currently is focusing on Safety and Risk Management at the Hadassah Medical Organization.
